# A human and animal model-based approach to investigating the anti-inflammatory profile and potential of the 5-HT_2B_ receptor antagonist AM1030

**DOI:** 10.1186/s12950-016-0127-2

**Published:** 2016-06-22

**Authors:** Niklas Palmqvist, Max Siller, Cecilia Klint, Anders Sjödin

**Affiliations:** AnaMar AB, R&D, Scheelevägen 2, SE-223 81 Lund, Sweden

**Keywords:** 5-HT, 5-HT_2B_ receptor, Inflammation, Immunomodulation, Dermatitis, AM1030

## Abstract

**Background:**

Atopic dermatitis (AD) is a chronic inflammatory skin disease characterized by highly pruritic eczematous lesions that are commonly treated with topical corticosteroids and calcineurin inhibitors. Side-effects and safety concerns associated with these agents restrict their use, and new, safe treatment options are therefore needed. Recent reports suggest that serotonin, i.e. 5-hydroxytryptamine (5-HT) and the 5-HT_2_ receptor family may contribute to inflammation and pruritus in the skin. The objective of this particular study was to investigate the 5HT_2B_ receptor antagonist AM1030 with respect to its anti-inflammatory profile and potential.

**Methods:**

AM1030 was tested in a set of distinct human and rodent in vitro and in vivo models, differing with respect to e.g. T cell involvement, triggering stimulus, main read-outs and route of drug administration. The in vitro systems used were staphylococcal enterotoxin A (SEA)-stimulated human peripheral blood mononuclear cells, lipopolysaccharide (LPS)-stimulated human primary monocytes, LPS-stimulated human THP-1 monocytes and LPS-stimulated mouse primary macrophages. The in vivo systems used were LPS- and SEA-induced cytokine production in the mouse, antigen-induced arthritis in the rat, glucose-6-phosphate isomerase-induced arthritis in the mouse and delayed-type hypersensitivity reaction in the mouse. In addition, different cell populations were analyzed with respect to their expression of the 5-HT_2B_ receptor at the mRNA level.

**Results:**

AM1030 significantly reduced both T cell-dependent and T cell-independent inflammatory responses, in vivo and in vitro. Due to the low or absent expression of the 5-HT_2B_ receptor on T cell populations, the influence of AM1030 in T cell-dependent systems is suggested to be mediated via an indirect effect involving antigen-presenting cell types, such as monocytes and macrophages.

**Conclusion:**

Based on the wide range of model systems used in this study, differing e.g. with respect to species, T cell involvement, triggering stimuli, route of drug administration and read-outs, our results suggest a broad anti-inflammatory effect of AM1030 and identify the 5-HT_2B_ receptor as a promising future target for anti-inflammatory intervention, e.g. in AD.

## Background

Atopic dermatitis (AD) is a common chronic inflammatory skin disease with substantial negative influence on the life quality of affected individuals. The central features of AD are skin dryness and eczematous lesions that are characterized by severe pruritus, erythema, excoriated/crusted papulovesicles, exudation and scaling, and in the chronic stage, skin thickening. The etiology of AD is complex and incompletely understood, but genetic predisposition in complex interplay with environmental factors is believed to be important [1, 2].

Skin barrier dysfunction and aberrant immune responses are key factors in the pathogenesis of AD [1, 3–5]. The acute stage of AD is typically dominated by a Th2-type of T cell response, believed to be triggered by environmental allergens [1, 4, 6]. The cutaneous hyperreactivity is fueled by the dysfunctional, leaky skin barrier, which increases the exposure of the immune system for allergens [1, 6, 7]. While the deficient skin barrier may amplify the cutaneous immune response, Th2 cytokines may also negatively influence the integrity of the epidermal barrier, e.g. by inhibiting the production of skin barrier proteins and antimicrobial peptides [3, 4]. In its chronic stage, the AD lesion is characterized by an immune response involving several T cell subsets, with a significant contribution of the Th1 subset [3, 6, 7]. This shift in T cell subset predominance may be caused by an increased exposure of the immune system to non-allergen triggers, including microbial products [6]. In line with this, skin colonization with superantigen-producing strains of *S. aureus* is very common in AD patients [6, 8, 9].

With respect to pharmacological treatment of AD, the dysregulated immune response is still the process mainly targeted by available therapies, e.g. topical corticosteroids and calcineurin inhibitors [2]. Although efficacious, side-effects and safety issues associated with these drugs limit their usefulness, especially as maintenance treatments. Thus, there is a need for new, safe anti-inflammatory/immunomodulatory agents that can be used for the induction and maintenance of clinical remission.

Several reports suggest that serotonin, i.e. 5-hydroxytryptamine (5-HT), first recognized as a vasoactive compound, plays a role in inflammation and immune responses as well as in pruritus, pain and fibrosis [10–21], all processes of pathophysiological relevance in dermatitis. For instance, several animal studies suggest the importance of 5-HT and 5-HT_2_ receptors for edema formation [12, 14, 19], T cell responsiveness [10, 16, 21] and scratching behaviour [22–25]. In humans, intradermal injection of 5-HT causes erythema, edema and, importantly, pruritus with rapid onset [20, 26]. In addition, 5-HT levels are increased in eczematous skin, e.g. in patients with allergic contact dermatitis (ACD) [27, 28]. Moreover, the pro-inflammatory role of the 5-HT_2_ receptor family in human skin is suggested by a clinical report showing the 5-HT_2_ receptor antagonist ketanserin to reduce ACD, a T cell-dependent reaction [29].

AM1030 is a novel 5-HT_2B_ receptor antagonist that displays binding to and functional inhibition of the human receptor (K_i_ = 0.33 μM; IC_50_ = 0.14 μM). Structurally, AM1030 is an aminoguanidine derivative related to a previously published compound with anti-inflammatory properties [30]. AM1030 is currently in clinical development phase.

Against this background, we initiated the current work, which had the objective to investigate AM1030 with respect to its anti-inflammatory profile and potential. To this end, a model-based approach was used, employing a set of distinct human and rodent in vitro and in vivo systems of relevance for a range of inflammatory diseases, including AD and ACD.

## Methods

### Human in vitro systems

#### Human peripheral blood mononuclear cells (PBMC)

Healthy donor blood was obtained from the Clinical Trials Unit, Skåne University Hospital, Lund, as approved by the Regional Ethical Review Board in Lund (Registration number: 2013/177). Peripheral blood mononuclear cells (PBMCs) were isolated by density gradient centrifugation (Ficoll® Paque Plus, GE Healthcare, Cat. no: 17-1440-02). The cells were suspended in cell culture medium consisting of RPMI 1640 with GlutaMAX™ (Gibco, Cat. no: 61870), 10 % Fetal bovine serum (FBS; Gibco, Cat. no: 10270), 100 U/ml penicillin, 100 μg/ml streptomycin (Gibco, Cat. no: 15140) and 2.5 μg/ml Fungizone® (amphotericin B, Gibco, Cat. no: 15290). Isolated PBMCs were added to 48-well culture plates (Corning, Costar®, Cat. no: 3548) at a density of 5 × 10^5^ cells/ml in the presence of 10 pg/ml staphylococcal enterotoxin A (SEA; Sigma-Aldrich, Cat. no: S9399) and drug compound (AM1030, the reference 5-HT_2B_ receptor antagonist RS127445 [31] (Tocris Bioscience, Cat. no: 2993) or the reference calcineurin inhibitor tacrolimus (FK-506; Tocris Bioscience, Cat. no: 3631)). The cell culture plates were incubated at 37 °C, 5 % CO_2_, in a humid environment. After 42 h incubation, the plates were centrifuged at 300 × *g* for 10 min. Supernatants were collected and cell viability was immediately assessed using the WST-1 reagent kit, in accordance with the manufacturer’s instructions (Roche, Cat. no: 11644807001).

#### Human primary monocytes

Human monocytes were purified from the PBMC fraction by negative selection using a pan monocyte isolation kit (Miltenyi Biotec, Cat. no: 130-096-537). The purified cells were suspended in cell culture medium consisting of RPMI 1640 with GlutaMAX™, 10 % heat-inactivated human AB serum (Sigma-Aldrich, Cat. no: H3667), 100 U/ml penicillin, 100 μg/ml streptomycin and 2.5 μg/ml Fungizone®. The cells were added to 96-well culture plates (Nunc™, Nunclon™ Delta surface, Cat. no: 167008) at a density of 1 × 10^5^ cells/well and the plates were incubated over-night (37 °C, 5 % CO_2_, humid environment). The next day, the culture medium containing non-adherent cells was removed and replaced with fresh medium containing 10 pg/ml lipopolysaccharide (LPS *Escherichia coli* 055:B5, Sigma-Aldrich, Cat. no: L6529) and drug compounds (AM1030 and RS127445). The plates were incubated for 4 h (37 °C, 5 % CO_2_, humid environment), after which supernatants were harvested and cell viability assessment was performed using the WST-1 reagent kit.

#### THP-1 monocytic cells

Human THP-1 monocytes (ATCC, Cat. no: TIB-202) were cultured at 37 °C, 5 % CO_2_ in RPMI 1640 with GlutaMAX™, 10 % heat-inactivated FBS and 50 μM β-mercaptoethanol (Sigma-Aldrich, Cat. no: M6250). Before the assay, cells were seeded at a density of 5 × 10^5^ cells/ml and pre-stimulated overnight with 10 U/ml interleukin 1-α (IL-1α; Sigma-Aldrich, Cat. no: I2778). At assay start, cells were seeded 2 × 10^5^ cells/well in a final volume of 200 μl. Compounds were added ~50 min before stimulation with 1.0 μg/ml of LPS. Supernatants for measurement of IL-6 were harvested 20 h after LPS stimulation. Cell viability was monitored using the WST-1 reagent kit. IL-6 content in cell supernatants was determined by ELISA.

### Animal in vitro systems

#### Mouse primary macrophages

The animal work was performed with permission from the local Ethical Committee on Animal Research, Malmö/Lund (permission no: M42-13). Mouse peritoneal macrophages were obtained by peritoneal lavage of female BALB/c mice (Taconic, Denmark) four days after intra-peritoneal injection of 1 ml 8 % thioglycolate in water. The lavage was performed after cervical dislocation, using 5 ml/mouse of culture medium without GM-CSF (see below). Cells were seeded in 96-well plates at 1 × 10^5^ cells/well in a total volume of 50 μl culture medium (RPMI 1640 with GlutaMAX™, 5 % heat-inactivated FBS, 50 μM β-mercaptoethanol, 100 U/ml penicillin, 100 μg/ml streptomycin and 2.5 μg/ml Fungizone®, 50 ng/ml mouse GM-CSF (Sigma-Aldrich, Cat. no: G0282). After 2 h incubation at 37 °C, 5 % CO_2_, non-adherent cells were removed by repeated washing with phosphate-buffered saline (Gibco, Cat. no: 14190). AM1030, 5-HT (1 μM; Sigma-Aldrich, Cat. no: 85036 Fluka) and LPS (25 ng/ml) were then added in a total volume of 200 μl. Supernatants were harvested after 20 h of incubation, and cell viability monitored using the WST-1 reagent kit. Supernatants were analyzed for IL-6 content using ELISA.

### Animal in vivo systems

#### LPS- and SEA-induced cytokine responses in mice

All experiments were approved by the local Ethical Committee on Animal Research, Malmö/Lund (permission nos: M140-09, M189-12). Female BALB/c (LPS model) and C57Bl/6 (SEA model) mice obtained from Taconic, Denmark were acclimatized for a minimum of one week prior to use in any experiments. The mice were pre-treated with drug compounds (AM1030, RS127445, tacrolimus, cyclosporine A (CsA; Tocris Bioscience, Cat. no: 1101)) or vehicle (as described in Figure legends) before intraperitoneal injection of LPS (0.5 mg/kg in normal saline) or SEA (0.5 mg/kg in normal saline). The animals were sacrificed 90 (LPS) or 120 (SEA) min after LPS/SEA injection and blood was collected for plasma analyses. Samples were stored at -20 °C until analysis.

#### Glucose-6-phosphate isomerase (G6PI)-induced arthritis in mice

All experiments were approved by the local Ethical Committee on Animal Research, Malmö/Lund (permission no: M155-10). Female DBA1 mice were obtained from Taconic, Denmark and were acclimatized for a minimum of one week prior to use in any experiments. To induce arthritis, animals were immunized subcutaneously with 200 μg (100 μL) rabbit glucose-6-phosphate isomerase (rabG6PI; Sigma-Aldrich, Cat. no: P9544) emulsified in Freund’s complete adjuvance (H37A, Difco, Cat. no: 263810). AM1030 (30 mg/kg) and vehicle (20 % Solutol® HS 15 (BASF, Cat. no: 51633963) in normal saline) were administered subcutaneously once daily, starting 4 days after immunization. Mice were visually scored for arthritis according to the following: each arthritic (red and swollen) toe and knuckle was scored as 1, whereas an affected ankle or wrist was scored as 5 (i.e. maximum score per paw: 15).

#### Antigen-induced arthritis (AIA) in rats

All experiments were approved by the local Ethical Committee on Animal Research, Malmö/Lund (permission nos: M229-06, M274-09). Female Dark Agouti rats were obtained from Harlan, The Netherlands and were acclimatized for a minimum of one week prior to use in any experiments. The rats were immunized subcutaneously at the tail root with 1 mg of methylated bovine serum albumin (mBSA; Sigma-Aldrich, Cat. no: A1009) dissolved in 50 μl normal saline and emulsified in 50 μl Freund’s complete adjuvance. Ten days later, each animal was subjected to an intra-articular challenge with antigen (75 μg mBSA in 50 μl normal saline) into the left knee joint. AM1030 was administered perorally or subcutaneously at the time of challenge, thereafter once daily for three consecutive days. Arthritis development was followed by daily measurements of knee swelling with an odometer/calliper (B2X048, Kroeplin Längernmesstechnik GmbH, Schlüchtern, Germany)*,* carried out under a brief isoflurane anaesthesia. The animals were sacrificed four days after challenge.

#### Delayed-type hypersensitivity reaction in mice

Oxazolone-induced delayed-type hypersensitivity (DTH) studies were performed by Eurofins Panlabs, Taiwan, as a contract research service. All aspects of the work were performed in accordance with the Guide for the Care and Use of Laboratory Animals (National Academy Press, Washington, D. C., 2011). Briefly, male BALB/c mice were sensitized by application of oxazolone (100 μL, 1.5 % in acetone) onto their shaved abdomen. Seven days later, AM1030 or vehicle (dipropylene glycol) was applied topically to the anterior and posterior surfaces of the right ear (20 μL/ear), 30 min before and 15 min after challenge with oxazolone (1 % in acetone, 20 μL/ear). After another 24 h, ear thickness was measured with a micrometer gauge. Ear swelling was calculated by subtracting the thickness of the left (control) ear from the right (treated) ear.

### Cytokine analyses

With the exception of mouse IL-17 (eBioscience, Cat. no: 88-7371-22), all other ELISA kits were from BD Biosciences: IFN-ɣ [Cat. nos: 555142 (human) and 551866 (mouse)], IL-2 [Cat. nos: 555190 (human) and 555148 (mouse)], IL-5 (Cat. no: 555202), IL-12 [Cat. nos: 555183 (human IL-12 p70) and 555165 (mouse IL-12 p40)], TNF [Cat. nos: 555212 (human) and 555268 (mouse)], IL-6 [Cat. nos: 555220 (human) and 555240 (mouse)].

### Real-time quantitative PCR (rt-qPCR) for HTR2B transcript analysis

Total RNA was isolated using RNeasy® Plus Mini Kit combined with QiaShredder™ and gDNA Eliminator Mini Spin Columns (Qiagen, Cat. nos: 74134, 79654 and 1030958, respectively). cDNA synthesis was performed using High-Capacity cDNA Reverse Transcription kit (Applied Biosystems, Cat. no: 4368814) or TaqMan® Reverse Transcription Reagents (Applied Biosystems, Cat. no: N8080234). A negative control reaction without reverse transcriptase enzyme was included for each cell type in every batch of cDNA synthesis. The rt-qPCR analysis was performed on a StepOnePlus™ instrument from Applied Biosystems, using 25 or 50 ng of cDNA template per well in a total reaction volume of 20 μL. Reference genes were assayed with the same amount of cDNA as the target genes. Amplification was achieved using primers/probes and MasterMix with Taqman® chemistry [Applied Biosystems, Universal Taqman® Gene Expression Master Mix (Cat. no: 4369016)] and inventoried recommended assays. Analysis of data was performed using StepOne™ Software v2.1. For details on primers/probes for 5-HT_2B_ receptor and reference gene analyses, see Table 1.

**Table 1:** Real-time quantitative PCR: Cell types, primers and results

Cell type	HTR2B primers	Reference gene primer	HTR2B expression
Human PBMCs	Taqman primer Hs00168362_m1	GAPDH: Taqman primer Hs02758991_g1	Very low or absent
(see Methods)			
		Hprt1: Taqman primer Hs02800695_m1	
		ActB: Taqman primer Hs1060665_g1	
		RPLP0: Taqman primer Hs004189669_g1	
Human T cell populations:	Taqman primer Hs00168362_m1	GAPDH: Taqman primer Hs02758991_g1	Very low or absent in all T cell subpopulations
-CD3+			
-CD4+			
-CD8+			
-CD4+ CD25+			
(total RNA obtained from Miltenyi Biotec; Cat. nos: 130-093-165, 164, 163 and 168)			
Human primary monocytes (see Methods)	Taqman primer Hs00168362_m1	GAPDH: Taqman primer Hs02758991_g1	Expressed
		Hprt1: Taqman primer Hs02800695_m1	
Human immature dendritic cells (total RNA obtained from 3H Biomedical; Cat. no: 3H100-70-5)	Taqman primer Hs00168362_m1	GAPDH: Taqman primer Hs02758991_g1	Expressed
		Hprt1: Taqman primer Hs02800695_m1	
		ActB: Taqman primer Hs1060665_g1	
		RPLP0: Taqman primer Hs00420895_gH	
Human THP-1 monocytes and macrophages (phorbol 12-myristate 13-acetate-differentiated)	Taqman primer Hs00168362_m1	GAPDH: Taqman primer Hs02758991_g1	Expressed under inflammatory conditions
		Hprt1: Taqman primer Hs02800695_m1	
		ActB: Taqman primer Hs1060665_g1	
		RPLP0: Taqman primer Hs00420895_gH	
Mouse peritoneal macrophages (thioglycolate-elicited, see Methods)	Taqman primer Mm00434123_m1	GAPDH: Taqman primer Mm99999915_g1	Expressed
		Hprt1: Taqman primer Mm00446968_m1	

## Results

### AM1030 reduces SEA-induced cytokine responses in human PBMCs

**Fig. 1:**
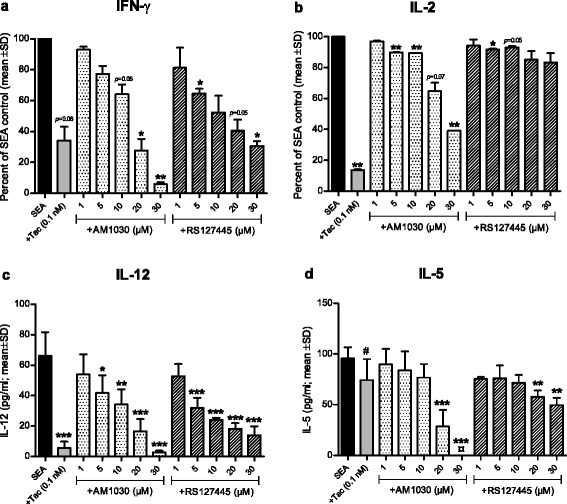
The effect of the 5-HT_2B_ receptor antagonist AM1030 on SEA-induced cytokine responses in human PBMCs. Tacrolimus (Tac) and RS127445 were included as reference drugs. IFN-ɣ (**a**) and IL-2 (**b**) data obtained from two healthy blood donors were pooled by normalizing sample cytokine levels to untreated control and are presented as percent of SEA (10 pg/ml) control. The absolute levels of IFN-ɣ and IL-2 were 9.6-24 ng/ml and 1.4-1.5 ng/ml, respectively. IL-12 (**c**) and IL-5 (**d**) data were obtained from a single blood donor and are presented as absolute values. One sample *t*-test (**a** and **b**) and one-way ANOVA with Dunnett’s post test (**c** and **d**) were used for statistical analysis (*p < 0.05, **p < 0.01, ***p < 0.001). ^#^At 1 nM, tacrolimus reduced IL-5 to background level. ^¤^At 30 μM, AM1030 reduced IL-5 to levels below detection

Staphylococcal enterotoxin A (SEA) is a T cell superantigen frequently expressed by *S. aureus* strains colonizing AD skin [8, 9]. In an in vitro model based on PBMCs isolated from healthy blood donors, SEA was used to mimic the process of antigen presentation and to thereby induce cytokine production from both antigen-presenting cells (APCs; e.g. macrophages) and T cells [32]. AM1030 dose-dependently inhibited SEA-induced production of IFN-ɣ, IL-2, IL-12 and IL-5 in PBMCs (Fig. 1). With the exception for IL-2, RS127445, the reference 5-HT_2B_ receptor antagonist showed similar, although less pronounced effects (Fig. 1). Tacrolimus, an immunosuppressive calcineurin inhibitor used in the treatment of AD, was included as a positive control. As expected, 0.1 nM tacrolimus was shown to inhibit the induction of all studied cytokines, with the exception of IL-5 (Fig. 1d), for which 1 nM tacrolimus was required (data not shown). Tacrolimus at 1 nM reduced all studied cytokines to levels approaching background (data not shown). Neither AM1030, RS127445 nor tacrolimus had any negative influence on the viability of the PBMCs (data not shown).

### AM1030 interferes with LPS-induced TNF and IL-6 production in human monocytes and mouse macrophages

Despite the inhibitory effect of AM1030 and RS127445 on SEA-induced cytokine production in human PBMCs, the presence of 5-HT_2B_ receptor mRNA transcript in the PBMC fraction could not be confirmed (Table 1). With T cells being the major cell type populating the PBMC fraction, this indicated that the demonstrated ability of AM1030 to interfere with SEA-induced production of T cell cytokines (i.e. IFN-ɣ, IL-2, IL-5) did not require the presence of the 5-HT_2B_ receptor in the T cell population. In line with this, the lack of detectable 5-HT_2B_ receptor transcript in different T cell populations was confirmed in an independent material (Table 1). These results together suggested that the 5-HT_2B_ receptor is not generally expressed at significant levels in T cells within the PBMC fraction. In contrast, 5-HT_2B_ receptor transcript was clearly present in adherent monocytes enriched from the PBMC fraction (Table 1), supporting that monocytes do express the receptor of interest at experimental conditions. Further support for the expression of the 5-HT_2B_ receptor within the APC fraction came from the rt-qPCR analysis of total RNA obtained from human immature dendritic cells (Table 1). Thus, cells within the APC fraction (e.g. monocytes, dendritic cells) may directly respond to the 5-HT_2B_ receptor antagonists and reduce their own production of cytokines. Indeed, this would explain the strong reduction of the typical APC cytokine IL-12 in response to the 5-HT_2B_ receptor antagonists in the PBMCs (Fig. 1c).

**Fig. 2:**
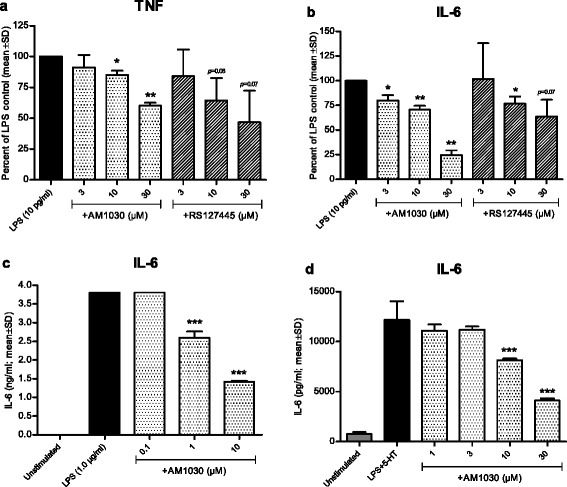
Influence of the 5-HT_2B_ antagonist AM1030 on LPS-induced cytokines in human monocytes and mouse macrophages. In (**a**) and (**b**), the effect of AM1030 and the reference 5-HT_2B_ receptor antagonist RS127445 on the production of TNF (**a**) and IL-6 (**b**) by LPS-stimulated human primary monocytes. Data resulting from three healthy blood donors were pooled by normalizing sample cytokine levels to untreated control and are presented as percent of LPS control. The absolute levels (mean ± SD) of TNF and IL-6 were 483 ± 13 pg/ml and 230 ± 84 pg/ml, respectively. In (**c**) and (**d**), the influence of AM1030 on the production of IL-6 by human THP-1 monocytes (**c**) and mouse peritoneal macrophages (**d**) is shown. One sample *t*-test (**a** and **b**) and one-way ANOVA with Dunnett’s post test (**c** and **d**) were used for statistical analysis (*p < 0.05, **p < 0.01, ***p < 0.001)

At this point, we hypothesized that the reduction of T cell responses shown with 5-HT_2B_ receptor antagonists in the PBMC system might be explained by a primary interference with monocyte/macrophage responses. For this reason, experiments on monocytes that had been purified from the PBMC fraction were performed. In the PBMC system monocytes can respond to SEA due to the presence of T cells in the culture. However, in the absence of T cells in the system, a T cell-independent stimulus such as LPS is required to trigger cytokine production. As shown in Fig. 2 (a-b), both AM1030 and RS127445 had dose-dependent effects on LPS-induced TNF and IL-6 in human primary monocytes. Our findings in primary human monocytes were further substantiated by additional observations showing that AM1030 suppressed IL-6 production in human THP-1 monocytes (Fig. 2c) and in primary mouse macrophages (Fig. 2d), both of which expressed the 5-HT_2B_ receptor at the mRNA level (Table 1). Neither AM1030 nor RS127445 had any negative influence on the viability of these cell types (data not shown).

### AM1030 reduces T cell-dependent and T cell-independent cytokine responses in mice

**Fig. 3:**
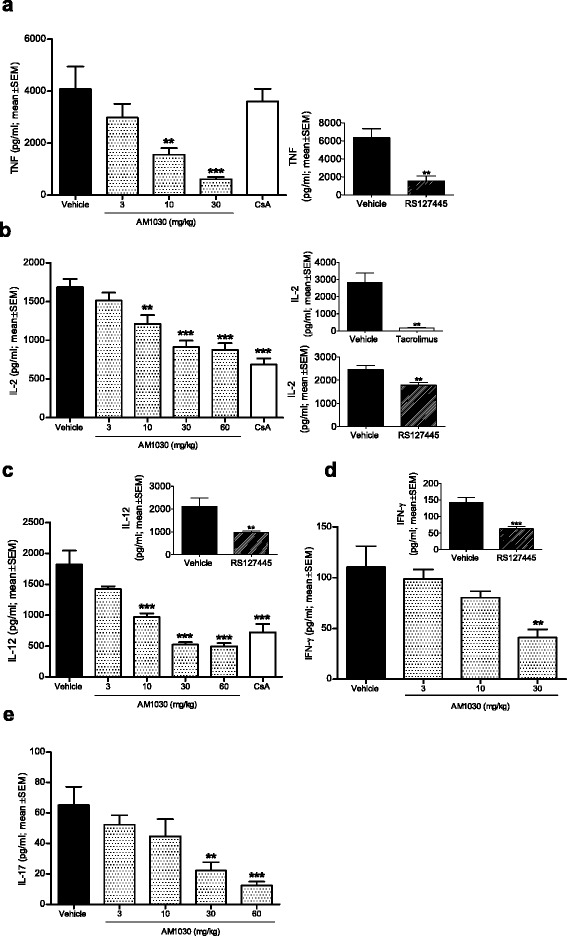
The effect of AM1030 and reference drugs on LPS- and SEA-induced cytokine responses in mice. **a** Effect of AM1030, CsA and RS127445 on LPS-induced TNF. AM1030 and CsA (30 mg/kg; *n* = 7-8/group) were administered subcutaneously 30 min before intraperitoneal injection of 0.5 mg/kg LPS (left). RS127445 (10 mg/kg; *n* = 8-9/group) was administered subcutaneously 15 min before LPS injection (right). TNF was analysed in plasma samples obtained 90 min after LPS injection. Vehicles were 5 % Kolliphor®EL, 20 % Tween 80 in normal saline (AM1030 and CsA) and normal saline (RS127445). **b** Effect of AM1030, CsA, tacrolimus and RS127445 on SEA-induced IL-2. AM1030, CsA (30 mg/kg; *n* = 7-8/group) and tacrolimus (3 mg/kg; *n* = 4/group) were administered subcutaneously 30 min before intraperitoneal injection of 0.5 mg/kg SEA. RS127445 (10 mg/kg; *n* = 8/group) was administered perorally 45 min before SEA injection. Vehicles were 5 % Kolliphor®EL, 20 % Tween 80 in normal saline (AM1030, CsA, tacrolimus) and 2.5 % Tween 80 in tap water (RS127445). **c** Effect of AM1030, CsA and RS127445 on SEA-induced IL-12. AM1030 and CsA (30 mg/kg; *n* = 7-8/group) were administered subcutaneously 30 min before intraperitoneal injection of 0.5 mg/kg SEA. RS127445 (10 mg/kg; *n* = 7-8/group) was administered perorally 45 min before SEA injection. Vehicles were 5 % Kolliphor®EL, 20 % Tween 80 in normal saline (AM1030 and CsA) and 2.5 % Tween 80 in tap water (RS127445). **d** Effect of AM1030 and RS127445 on SEA-induced IFN-ɣ. AM1030 was administered subcutaneously 30 min before intraperitoneal injection of 0.5 mg/kg SEA (*n* = 8-10/group). RS127445 was administered perorally 45 min before SEA injection (10 mg/kg; *n* = 6-8/group). Vehicles were 10 % Tween 80 in normal saline (AM1030) and 2.5 % Tween 80 in tap water (RS127445). **e** Effect of AM1030 on SEA-induced IL-17. AM1030 was administered subcutaneously 30 min before intraperitoneal injection of 0.5 mg/kg SEA (*n* = 7-8/group). The vehicle was 5 % Kolliphor®EL, 20 % Tween 80 in normal saline. One-way ANOVA with Dunnett’s post-test and unpaired *t*-test were used for statistical analyses as appropriate (**p < 0.01, ***p < 0.001)

To further investigate the anti-inflammatory/immunomodulatory properties of AM1030, the compound was evaluated in two different in vivo systems in the mouse. Systemic injection of either SEA or LPS was utilized to induce T cell-dependent and T cell-independent cytokine responses, respectively. The T cell-independence of the LPS-induced cytokine response was indicated by the absence of effect of CsA (Fig. 3a), whereas CsA as well as tacrolimus, both calcineurin inhibitors, proved efficacious in the T cell-dependent SEA-model (Fig. 3b-c). As shown in Fig. 3, systemic treatment with AM1030 dose-dependently reduced the production of cytokines in both these systems, leading to lower plasma levels of TNF, IL-2, IFN-ɣ, IL-12 and IL-17. The reference 5-HT_2B_ receptor antagonist RS127445 also reduced cytokine production in these different systems (Fig. 3a-d).

### AM1030 ameliorates arthritis in rats and mice

**Fig. 4:**
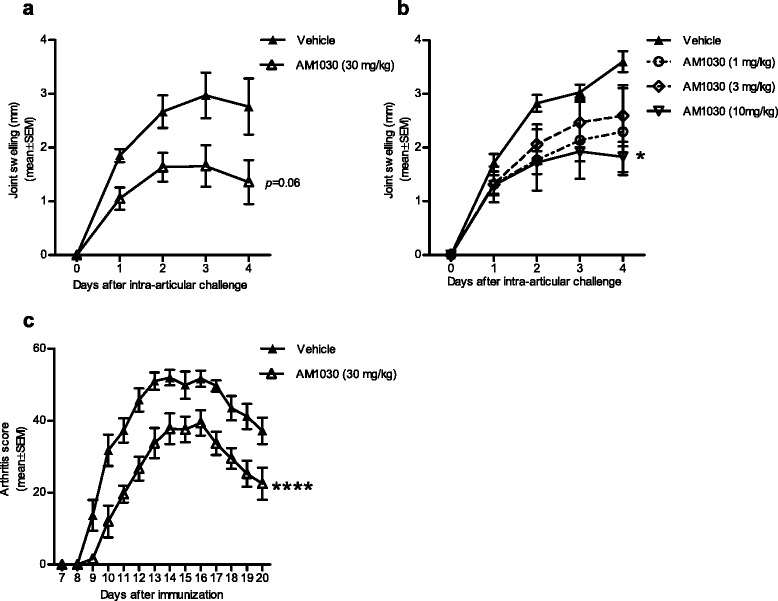
The effect of systemic treatment with AM1030 in murine arthritis models. Systemic treatment with AM1030 reduced arthritis development in rat AIA (**a**, **b**) and mouse G6PI-induced arthritis (**c**) as compared to vehicle. In (**a**), once daily subcutaneous treatment with 30 mg/kg AM1030 (*p* = 0.06 vs. vehicle (20 % Solutol® HS15 in normal saline), *n* = 6/group). In (**b**), once daily peroral treatment with 1-10 mg/kg AM1030 in water (*p < 0.05 at 10 mg/kg, *n* = 6/group). In (**c**), once daily subcutaneous treatment with 30 mg/kg AM1030 (****p = 0.0001 vs. vehicle (20 % Solutol® HS15 in normal saline), *n* = 10/group). Joint swelling and arthritis score data obtained from each individual animal were summed for the whole evaluation period, after which the Mann-Whitney test (**a**, **c**) or the Kruskal-Wallis one-way ANOVA with Dunn’s post test (**b**) were used for statistical analysis

AIA in the rat and G6PI-induced arthritis in the mouse are both T cell-dependent arthritis models that require TNF and IL-6 [33–38] for arthritis development. Due to the involvement of both T cells and APCs (e.g. TNF-producing macrophages) in these models, they seemed appropriate for the further evaluation of AM1030. As shown in Fig. 4, the results obtained from these models were overall positive, with once daily systemic administration of AM1030 significantly ameliorating joint swelling and arthritis severity in AIA and G6PI-induced arthritis, respectively. However, pharmacokinetic investigations done in parallel indicated a rapid systemic elimination of the compound in rodents (data not shown). Thus, despite the efficacy of systemically administered AM1030 shown in rodent disease models, the pharmacokinetic profile of the compound spoke in favour of a non-systemic approach to treatment.

### Topical administration of AM1030 decreases ear swelling in an oxazolone-induced DTH reaction

**Fig. 5:**
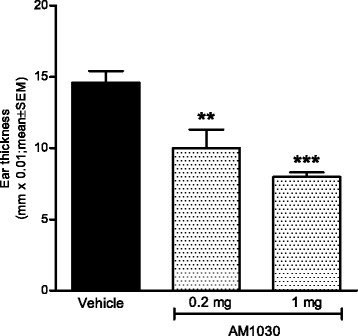
The effect of topically applied AM1030 on oxazolone-induced DTH reaction. Topical administration of AM1030 (0.2 and 1 mg) in vehicle (dipropylene glycol) significantly reduced ear swelling in the oxazolone-induced DTH reaction in the mouse. AM1030 or vehicle was applied to the anterior and posterior surfaces of the right ear (20 μL/ear), 30 min before and 15 min after challenge with oxazolone. Ear swelling responses were measured 24 h later. One-way ANOVA with Dunnett’s post test was used for statistical analysis (**p < 0.01, ***p < 0.001; *n* = 5/group)

Due to the pharmacokinetic profile of AM1030, which indicated a rapid systemic elimination in rodents, alternative non-systemic routes of administration were considered. Topical administration opens for the treatment of various inflammatory skin diseases, including AD. We therefore employed topical treatment in the oxazolone-induced DTH model, a type IV hypersensitivity reaction with mechanistic resemblance to ACD as well as AD [39]. As shown in Fig. 5, topical treatment with AM1030 significantly reduced oxazolone-induced ear swelling.

## Discussion

In the current report a set of different in vitro and in vivo model systems was used to investigate the 5-HT_2B_ receptor antagonist AM1030 with respect to its anti-inflammatory potential and profile. Based on the diversity of the employed model systems, e.g. with respect to activating triggers (SEA, LPS, recall antigen), responding cell populations (T cells, APCs, monocytes, macrophages), species (human, mouse and rat), read-outs (T cell cytokines, APC/monocyte/macrophage cytokines, in vivo inflammatory reactions) and drug administration routes (peroral, subcutaneous, topical), we conclude that AM1030 has broad anti-inflammatory and immunomodulatory effects.

The use of human PBMCs in combination with SEA enables us to mimic certain aspects of antigen presentation [32], a process of general significance in inflammatory diseases, including AD. In this in vitro system, AM1030 was demonstrated to reduce the production of cytokines from both adaptive (IL-2, IFN-ɣ, IL-5) and innate (IL-12) immune cells (Fig. 1). Our initial observations in human PBMCs were corroborated by in vivo findings in mice, in which AM1030 was able to suppress the SEA-induced cytokines of interest (Fig. 3b-e). The use of SEA as stimulus in our models substantiates the relevance of our findings in relation to AD, since AD patients are commonly colonized with superantigen-producing strains of *S. aureus* [8, 9]. By using LPS, a T cell-independent stimulus, in vitro and in vivo, we were then able to show the effect of AM1030 on monocyte/macrophage responses (Figs. 2 and 3a).

In summary, our combined pharmacological data and 5-HT_2B_ receptor expression data (mRNA; Table 1) suggest that the immunomodulatory effect of AM1030 in T cell-dependent reactions depends on its primary influence on cell types within the APC subset, such as monocytes and macrophages. The mechanistic basis for this influence remains to be investigated. Importantly, our results suggest that 5-HT_2B_ receptor signaling has impact on cytokine production by APCs, triggered by both MHC class II (SEA) and toll-like receptor 4 (LPS) stimulation. Therefore, follow-up studies should focus on identifying common denominators in intracellular signaling pathways shared by the 5-HT_2B_ receptor, MHC class II and toll-like receptor 4. The extracellular signal-regulated kinase (Erk) pathway is a possible candidate and might be a good starting point for such investigations, since it has been described to be involved in both 5-HT_2B_ receptor-induced signaling [40] and LPS-induced pro-inflammatory cytokine production in monocytes [41, 42]. Moreover, MHC class II activation has been shown to induce sustained Erk activity in APCs [43]. With regard to the role of Erk in LPS-induced cytokine responses, our own unpublished data clearly show that inhibition of the Erk pathway with two distinct MEK (Erk kinase) inhibitors reduces TNF and IL-6 production in LPS-stimulated THP-1 cells. Follow-up studies should also address whether the 5-HT_2B_ receptor might regulate gene transcription independently from additional triggers.

The indirect effect of 5-HT_2B_ receptor activation on T cell responses that is suggested by our data should also be further addressed in future studies. Currently, one plausible explanation supported by the literature is that APC-derived pro-inflammatory cytokines, e.g. TNF, IL-6 and IL-12, can provide additional signals to enhance T cell activation [44, 45].

The list of pathogenic mediators in AD and candidate target molecules for disease intervention is continuously growing. There are currently a few biological approaches to treatment underway that hold promise for the future, e.g. dupilumab, targeting the shared IL-4Rα subunit, thus interfering with both IL-4 and IL-13 signaling [46], and ustekinumab, targeting the p40 subunit shared by IL-12 and IL-23 [47, 48]. However, previous attempts to neutralize single mediators in AD have been rather disappointing, e.g. anti-IL-5 [49] and anti-IgE [50, 51], presumably due to the redundancy of disease-driving mediators combined with the heterogeneous nature of the disease. An alternative approach towards new treatment is the development of small molecular drugs that, rather than neutralizing a single mediator, have potential to simultaneously target several different pathogenic cell types and associated downstream processes. The relevance of this approach is shown by the well-established efficacy of topical corticosteroids and calcineurin inhibitors, having in common their broad immunomodulatory and anti-inflammatory effects [2].

The anti-inflammatory and immunomodulatory effects of AM1030 and RS127445 reported herein support the concept of using 5-HT_2B_ receptor antagonists for the treatment of inflammatory diseases, including AD. This concept is also supported by earlier studies investigating the role of 5-HT and the 5-HT_2_ receptor family in human and animal systems, addressing various aspects of inflammation, including pruritus [10–27, 29]. Whether a rapid onset anti-pruritic effect could be achieved by topical administration of a 5-HT_2B_ receptor antagonist is currently unknown and beyond the scope of this paper. However, having in mind the questioned efficacy of anti-histamines in AD [1], a 5-HT_2B_ receptor antagonist drug with rapid, yet sustained anti-pruritic and anti-inflammatory effects would be a welcome addition to the current treatment arsenal. Studies in humans will of course be necessary to explore the full potential of 5-HT_2B_ receptor antagonists in pruritic dermatitis.

The presented in vivo results show that AM1030, a 5-HT_2B_ receptor antagonist, has broad anti-inflammatory/immunomodulatory effects after systemic administration in different animal models (Figs. 3 and 4). However, in the course of our work, new information obtained from pharmacokinetic studies indicated a rapid systemic elimination of AM1030, which made us consider non-systemic routes of administration, such as topical application onto the skin. Interestingly, topically applied AM1030 reduced the oxazolone-induced DTH reaction in mice (Fig. 5), a T cell-dependent type IV hypersensitivity reaction with mechanistic resemblance to ACD as well as AD [39]. Considering the reported ability of the 5-HT_2_ receptor antagonist ketanserin to reduce ACD in humans [29], our DTH results seem rather encouraging.

## Conclusions

In conclusion, the 5-HT_2B_ receptor antagonist AM1030 has therapeutic potential in various inflammatory diseases, which is due to its inhibition of both T cell-dependent and T cell-independent responses. However, the pharmacokinetic profile of AM1030 probably makes it particularly suitable for topical treatment. In line with this, a first-in-man study in AD patients has recently been completed, supporting the safety and tolerability of topical treatment with AM1030.
